# A Long-term Survival Risk Prediction Model for Patients with Superficial Esophageal Squamous Cell Carcinoma

**DOI:** 10.7150/jca.99042

**Published:** 2024-10-14

**Authors:** Ruoyun Yang, Min Wei, Xin Yu, Wei Su, Xiaoying Zhou, Han Chen, Guoxin Zhang

**Affiliations:** 1 Department of Gastroenterology, The First Affiliated Hospital of Nanjing Medical University, Nanjing, China.; 2 The First Clinical Medical College, Nanjing Medical University, Nanjing, China.; 3 Department of Gastroenterology, Nanjing Jiangning Hospital, Nanjing, China.

**Keywords:** Superficial esophageal squamous cell carcinoma (SESCC), Nomogram, Prediction model, Emoglobin (Hb), Alpha-Fetoprotein (AFP)

## Abstract

**Objectives**: Given the data regarding the long-term prognosis of superficial esophageal squamous cell carcinoma (SESCC) is still lacking, we aimed to identify reliable prognostic factors and establish a high-precision prognosis model for patients with SESCC.

**Methods**: A retrospective cohort study was conducted including patients with SESCC at a high-volume tertiary medical center. The primary outcome was disease-specific survival (DSS) at the end of follow-up (minimum of 29 months). Independent prognostic factors including innovative hematological and clinicopathological parameters were identified using comprehensive and novel statistical methods including best subset regression (BSR), the univariate and multivariate Cox analysis, lasso regression, and a dynamic nomogram model was established.

**Results**: A total of 1,171 patients were finally enrolled. The median follow-up time is 83 months (range 29-149 months). Ten independent prognostic risk factors for a poor DSS were identified as follows: male (*P=*0.127), higher Charlson Comorbidity Index (CCI) (*P=*0.006), poorly differentiated tumor (*P*<0.001), lymphovascular invasion (LVI) (*P*<0.001), lymph node metastasis (LNM) (*P*<0.001), additional treatment (*P*=0.007), neutrophils over 32.2x10^9^/L (*P*=0.003), red blood cell (RBC) lower than 4.45x10^12^/L (*P*<0.001), hemoglobin (Hb) lower than or equal to 98 g/L (*P*=0.023), alpha-fetoprotein (AFP) higher than 3.24 ng/ml (*P*=0.034). Subsequently, an online dynamic nomogram was established *(https://yryouzu-tools.shinyapps.io/DynNomapp/)*. This prediction model showed favourable discrimination ability (area under the curve (AUC) was 0.913 (95% CI: 88.0 - 94.6) and a well-fitted calibration curve.

**Conclusions**: We successfully established a long-term prognosis model for SESCC, which can be applied to effectively predict survival risks for patients, thus strengthening follow-up strategies.

## Introduction

Esophageal cancer ranks as the 8th most prevalent cancer 1 and the 6th leading cause of cancer-related death worldwide^2^, thus representing a major global health challenge^3^. Esophageal cancer presents two distinct histological types: Adenocarcinoma (AC) and Squamous Cell Carcinoma (SqCC), with the latter exhibiting greater aggressiveness and a more unfavorable prognosis^4^. Superficial esophageal squamous cell carcinoma (SESCC) is defined as pathology-confirmed squamous cell carcinoma (SCC) that originated from the esophageal mucosa or submucosa, regardless of lymph node metastasis^4^.

Currently, the long-term prognosis of SESCC is still lacking. Moreover, there is no special and effective standard for predicting SESCC prognosis and guiding therapy. Therefore, a user-friendly and meaningful statistical prediction nomogram is needed for determining the prognosis for SESCC. Some nomograms have been reported in esophageal squamous cell carcinoma^5^ and adenocarcinoma^6^, two main types of esophageal cancer. However, to our knowledge, there is no prognostic prediction nomogram reported specifically for SESCC.

Cancer-related systemic inflammation has been shown to play a crucial role in the diagnosis and prognosis of cancers, especially in early cancers. A nomogram based on nutrition- and inflammation-related indicators to predict small cell carcinoma of the esophagus outcomes has been reported^7^. Our previous studies also found hematological parameters of monocyte-to-lymphocyte ratio (MLR) and Platelet Distribution Width (PDW) can be used as an adjuvant tool for the diagnosis of early esophageal cancer^8^ and a multi-analyte panel consisted of Hematocrit (HCT), Activated Partial Thromboplastin Time (APTT), Retinol-Binding Proteins (RBP), and Mean Platelet Volume (MPV) was able to discern the preoperative lymph nodal status of SESCC patients^9^. Therefore, we speculated that hematological parameters may serve as a simple tool for predicting the prognoses of patients with SESCC. In order to make the nomogram more comprehensive and efficient, we included the routine blood test (RBT) along with certain well-known risk factors of SESCC and constructed a dynamic nomogram to help predict outcomes in clinical work.

## Materials and Methods

### Patients and study design

**Figure 1:**
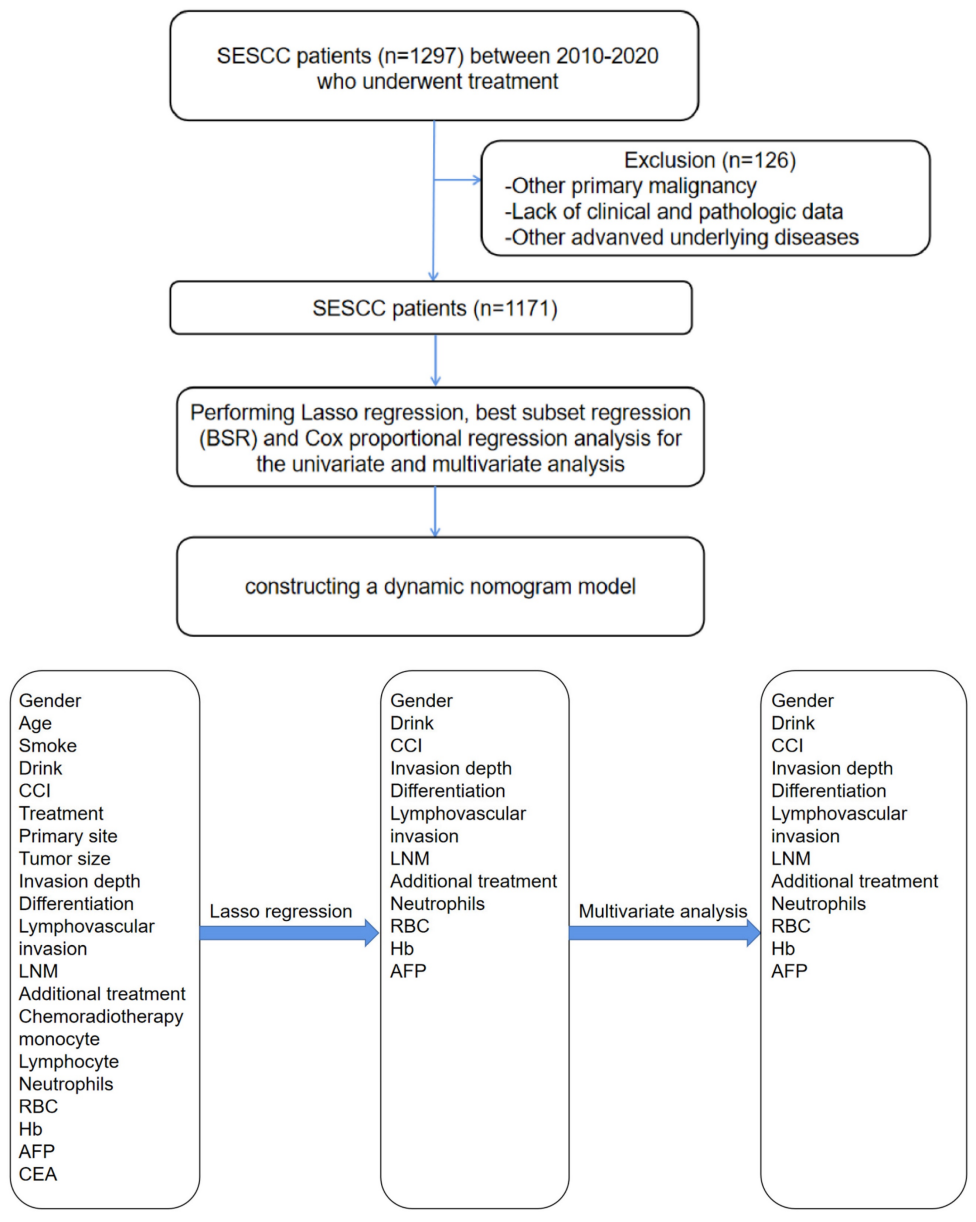
** (A) (B): The workflow of this study.** SESCC = superficial esophageal squamous cell carcinoma.

A retrospective cohort study was conducted including patients with SESCC who underwent treatment (including esophagectomy or endoscopic treatment (ED)) at a high-volume tertiary medical center from 2010 to 2020 (**Figure 1A**). From January 2010 to February 2020, data were collected among SESCC patients who have already received treatment and meet the follow-up time criteria (treatment over 6 months). All patient underwent endoscopic assessment, which included chromoendoscopy utilizing Lugol's dye spray method. Additionally, the majority of patients underwent EUS to ascertain if the cancer was limited to the mucosa. Furthermore, chest CT and/or positron emission tomography-CT scans were conducted to detect potential distant metastases or LNM. After completing the initial treatment phase, a rigorous follow-up protocol was implemented to monitor patient outcomes and gather longitudinal data. Patients were scheduled for regular follow-up visits at intervals of 3 months during the first year post-treatment, followed by bi-annual visits in subsequent years by telephone. Each follow-up visit included a comprehensive clinical assessment, including LNM, additional treatment, recurrence/metastasis and survival time. The follow-up period extended beyond 6 months post-treatment to capture long-term survival outcomes and late treatment effects. This structured follow-up approach ensured continuity of care and facilitated the collection of robust data for ongoing analysis and refinement of treatment protocols.

This study was conducted in accordance with the Helsinki Declaration and the protocol was approved by the institutional review board of the First Affiliated Hospital of Nanjing Medical University (2022-SR-370). This study was registered in the Chinese Clinical Trial Registry (ChiCTR) website (ChiCTR2200064868).

### Data collecting procedure

Inclusion criteria included: (1) diagnosis of esophageal squamous carcinoma based on biopsy pathology; (2) pT1 stage carcinoma (no tumor invasion beyond the submucosa); (3) esophageal cancer was the primary malignancy, with only one primary tumor present; (4) the survival status and survival duration were clearly determined. Exclusion criteria included: (1) all non-squamous cell lesions of the esophagus (including Barrett's esophagus, etc.); (2) a mix of other subtypes of esophageal cancer; (3) tumor of uncertain pathological origin or esophageal cancer with metastasis; (4) patients under 18; (5) accompanied by other severe underlying diseases, such as heart disease, respiratory failure, severe renal or hepatic dysfunction.

Overall survival (OS) and DSS were defined as time from date of diagnosis to any form of mortality, and time from date of diagnosis to any form of recurrence or mortality specifically related to SESCC, respectively. Before surgery, all participants underwent histopathological evaluations following endoscopic biopsies to define esophageal cancer. LNM in CT was defined as the presence of at least one enlarged lymph node with a short-axis dimension of ≥1cm. LVI in Immunohistochemistry refers to the infiltration or penetration of blood vessels or lymphatic vessels by cancer cells. Additional treatment refers to supplemental therapy after primary treatment, including ER and surgery. Recurrences were classified as locoregional or distant according to the first relapse pattern. Locoregional recurrences were defined as recurrences within esophagus or regional lymph nodes, whereas distant recurrences were defined as nonregional lymph node recurrences (supraclavicular or para-aortic nodes), peritoneal carcinomatosis, or systemic metastases. Recurrences were established on histologic, cytologic, or explicit radiologic proof in each center. It includes metachronous esophageal squamous cell carcinoma.

The 8th edition AJCC/UICC staging system of esophageal cancer was applied^10^. Lesion diameters were determined as the maximum diameter in two dimensions, measured using Vernier's calipers. Tumor location was defined as the position of the epicenter of tumor. In cases where epicenter statement was not provided, measurements were approximated as: (1) upper: 15-24cm from incisors; (2) middle: 25-29cm from incisors; (3) lower: 30-40/45cm from incisors. Histologic grade (G) was categorized as well-differentiated (G1), moderately differentiated (G2) and poorly differentiated (G3). Macroscopic tumor type was classified following the 2016 Japanese Classification of Esophageal Cancer, 11th Edition^11^. General clinical features were recorded. A history of alcohol taking means consuming at least 60g of ethanol per day for men and at least 40g for women within the past five years of cancer diagnosis, as defined by WHO and the European Medicines Agency^12^. Two experienced pathologists who independently assessed the surgically resected specimens conducted the pathologic diagnosis of esophageal squamous cell carcinoma. Patients with pathologic findings indicating tumor invasion depth of T1b-SM1 or greater, presence of lymphovascular invasion, or positive resection margins were recommended for additional treatment, such as radical esophagectomy. Alternatively, patients who preferred to preserve their esophagus underwent ER. The patient's overall health condition was thoroughly assessed to determine suitability for additional treatment. Invasion depth was categorized into four categories: M1 (confined to the intraepithelium), M2 (confined to the lamina propria), M3 (confined to the muscularis mucosa) and submucosal (SM1, SM2 or deeper), which was further confirmed by immunohistochemical staining.

### Statistical analysis

The sample size was calculated by Cox regression. Quantitative data were presented as mean ± standard deviation (SD) and intergroup differences were analyzed by *Student's t tests*. Categorical data were described as counts and percentages (N, %). The difference between groups was analyzed using chi-square tests. We generated the 1:1 matched survival group and deceased group using a propensity score matching (PSM) method to reduce the effects of differences in baseline features. Age, gender, treatment, smoke, alcohol were included for matching. Some clinical and demographic continuous variables such as age and routine blood test were converted into categorical variables by using the X-tile program^13^. Lasso regression, BSR and Cox proportional regression analysis were performed for the univariate and multivariate analysis of prognostic factors, including: gender, age, smoke, alcohol, treatment, CCI, primary site, invasion depth, differentiation, LVI, LNM, additional treatment, chemoradiotherapy, monocyte, neutrophils, lymphocyte, RBC, Hb, AFP, carcinoembryonic antigen (CEA). The procedure and standard of blood assessment were described in our previous study^9^. Statistical significance was set at alpha=0.05, two-sided. Statistical analyses were conducted by the SPSS 22.0 and R software version 3.6.2.

### Model establishment

Specifically, univariate Cox regression, BSR and lasso regression with cross-validation were first utilized to screen latent risk factors. Subsequently, the variables selected by these three methods were included in the multivariate Cox regression (**Figure 1B**), performing a stepwise backward regression to determine the final set of variables based on the minimum Akaike Information Criterion (AIC) value. The nomograms were constructed based on these three methods using the “rms” package and then compared by receiver-operating characteristics (ROC) curves. The one with the biggest AUC was chosen as the optimal model for constructing the nomogram. The nomogram model validation was performed by AUC and the concordance index (C-index) for discrimination ability, and calibration curves for calibration. Bootstraps with 1,000 resamples were adopted to decrease the overfit bias. The nomogram was utilized in an internal validation cohort to further evaluate its robustness.

## Results

### Patient demographics

A total of 1171 SESCC patients, among whom 90 patients died of SESCC at last were identified. **Table 1** summarized the clinical characteristics of these two groups before and after PSM. A larger proportion of patients who died of SESCC experienced esophagectomy (80.0% versus. 33.5%, *P*<0.001) and had a higher CCI (*P*<0.05). Distribution of gender, history of smoking and drinking were similar between the two groups. After propensity score matching (PSM), the baseline characteristics of patients who alive and died of SESCC had no significant differences.

In terms of the pathologic features, patients who died from SESCC had lower and whole location cancer (all <0.05). The patients who died of SESCC had more T1a-m3 and T1b cancer (all <0.05). After PSM, there was no difference of lesion location between the two groups and the deceased group still had more T1b cancer (*P*<0.001). With regard to the tumor differentiation, the surviving patients had more well differentiated tumors (*P*<0.001). After PSM, the difference still existed (P<0.001). Both before and after PSM, there were more patients who had lymphovascular invasion and LNM in the deceased group (*P*<0.001).

In terms of adjuvant therapy, 113 living patients and 11 patients with poor survival received additional treatment for esophageal cancer including repeated ESD or esophagectomy (10.5% versus. 12.2%, *P*=0.730). After PSM, the difference no longer existed.

### Identification of independent risk factors and establishment of the dynamic nomogram for predicting the long-term survival of SESCC

We used three algorithms (univariate Cox regression, BSR and lasso regression) to screen potential prognostic factors. First, the variables included in the prediction model based on univariate Cox regression were as follows: gender, drink, LVI, LNM, differentiation, CEA, monocyte, with an AUC of 91.0 (87.7, 94.3). Second, the variables included in the prediction model based on BSR were as follows: gender, smoke, LVI, LNM, tumor size, differentiation, invasion depth, RBC, AFP, Hb, with an AUC of 89.9 (86.8, 93.1). Third, the variables included in the prediction model based on lasso regression were as follows: gender, CCI, differentiation, LVI, LNM, additional treatment, neutrophils, RBC, Hb, AFP, with an AUC of 91.3 (88.0, 94.6). According to the statistical methods described above, we selected the lasso regression algorithm with the highest AUC, so the final predictive candidates included in the nomogram were as follows: male (*P*=0.127), CCI (*P*=0.006), poorly differentiated tumor (*P*<0.001), LVI (*P*<0.001), LNM (*P*<0.001), additional treatment (*P*=0.007), neutrophils over 32.2x10^9^/L (*P*=0.003), RBC lower than 4.45x10^12^/L (*P*<0.001), Hb lower than or equal to 98 g/L (*P*=0.023), AFP higher than 3.24 ng/ml (*P*=0.034). We also establish the nomogram for OS, which can be reviewed in the **Supplementary Figure 1**. An online version of the nomogram was available *(https://yryouzu-tools.shinyapps.io/DynNomapp/).*

### Model performance and validation

**Figure 2:**
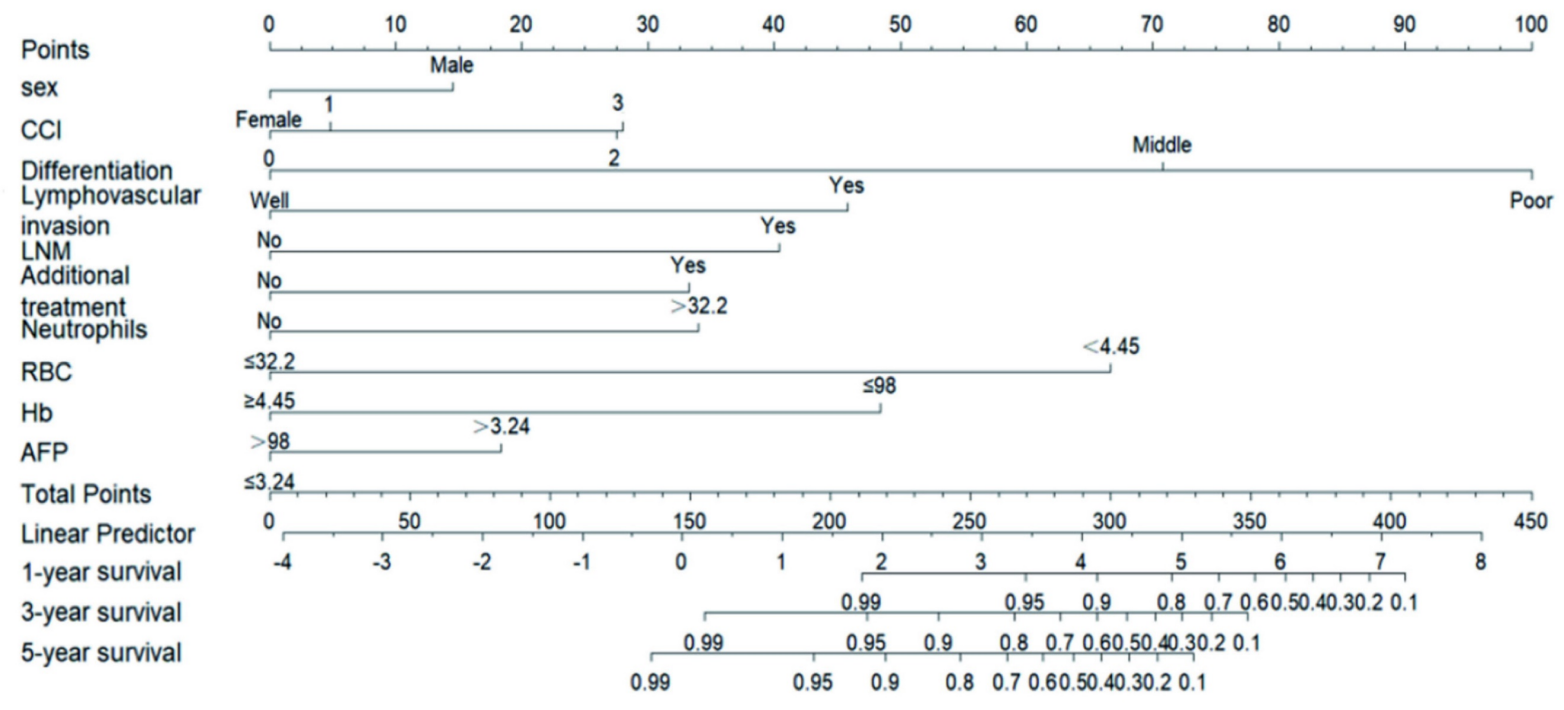
** Nomogram for the early prediction the SESCC free survival probability.** Characteristics in the nomogram to predict probability of SESCC free survival. To use the nomogram, the specific points of individual patients are located on each variable axis. Lines and dots are drawn upward to determine the points received by each variable; the sum of these points is located on the Total Points axis, and a line is drawn downward to the “1-year survival, 3-year survival and 5-year survival” axes to determine the probability of SESCC free survival. CCI = Charlson Comorbidity Index; LNM = lymph node metastasis; RBC = red blood cell; Hb = haemoglobin; AFP = Alpha-Fetoprotein.

**Figure 3:**
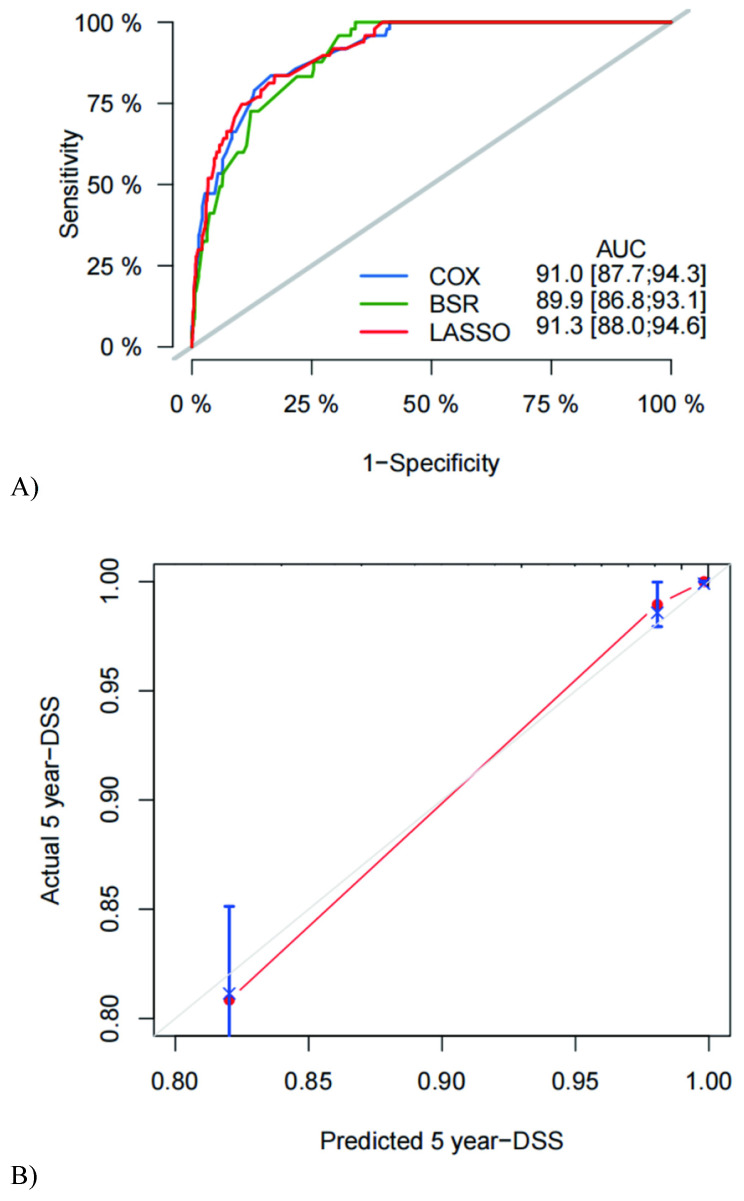
** ROC curve, AUC (A), and calibration curve (B) for DSS of the nomogram.** ROC = receiver operating characteristic; AUC = the area under ROC curve; DSS = disease-specific survival. Calibration curves of 5 years SESCC free survival.

The C-index was used to evaluate nomogram discrimination, which enumerates the level of concordance between the predicted and observed DSS or OS. The C-indexes of the nomograms in predicting DSS and OS were respectively 0.876 (95% CI: 0.849~0.903) and 0.878 (95% CI: 0.854~0.903). The prediction results of the 1-, 3- and 5-year DSS and OS rates are shown in **Figure 2 and Supplementary Figure 1**. The ROC curves and AUC indicated that the models have a good discrimination ability (**Figure 3A and Supplementary Figure 2A,** AUC were 0.913 for DSS and 0.910 for OS). Furthermore, the calibration curves of the DSS and OS rates showed the models fit well (**Figure 3B and Supplementary Figure 2B**). A total of 820 subjects were also included in the internal validation set. The results of the validation group were in accordance with the primary group (**Supplementary Figure 3**).

## Discussion

SESCC is a separate type of esophageal cancer. The traditional TNM staging system is used to predict the survival of esophageal cancers, but is not specific for SESCC. A more feasible survival predictive model is needed. In this cohort study, we screened the clinicopathological characteristics and pretreatment hematological parameters of SESCC patients who went to our hospital to perform treatment and explored independent risk factors in the prognosis of SESCC. Due to the absence of sufficient data on the long-term prognosis of SESCC, we successfully identified dependable prognostic factors and developed a high-precision model for the long-term prognosis of patients with SESCC.

Some common risk factors in accordance with the previous studies were screened out. The degree of differentiation served as a prognostic factor in many cancers and the more severe the tumor differentiation, the worse the prognosis^14^,^15^. Here in our study, we also reported a similar result. At present, the pathological stage is still one of the primary factors for determining the prognosis of patients with SESCC^16^,^17^, and our results also revealed a worse prognosis in patients with higher grades. There are also other factors that have a certain impact on the prognosis, such as LVI and additional treatments^18^-^20^.

Additionally, we found some innovative results. Different from the previous study^18^, our study found that the DSS of SESCC patients receiving additional ESD or esophagectomy get worse compared with those who did not need additional treatment. This may due to the fact that these patients had more severe disease-specific conditions which needed intervention of invasive operations that could lead to complications or impair their postoperative quality of life. Apart from the well-known risk factors, we included the innovative factor - CCI. We found patients who had more comorbidity turned out to have a poorer DSS. In Charlson's review, they also confirmed the CCI can be used to predict long-term mortality in different clinical populations, including medical, surgical, intensive care unit (ICU), trauma, and cancer patients^21^. By adding CCI to other measures in our predicted model increases the overall predictive accuracy.

Moreover, we discovered the potential value of hematological parameters in predicting the prognosis of SESCC. Hematological parameters in the noninvasive RBT have traditionally served as indicators of systemic inflammatory response, which has been found to be related to cancer development through genotoxicity, aberrant tissue repair, proliferative responses, invasion and metastasis^22^,^23^. Based on our previous research findings 8, we found they also functioned in the prognosis of SESCC, which confirmed our hypothesis.

Neutrophils contribute to tumor angiogenesis by generating proangiogenic factors, promoting the adhesion and seeding of distant sites 24. Neutrophilia can hinder the immune system by suppressing the cytolytic effects of immune cells 25. Besides, hemoglobin and RBC serve as crucial nutrition-related prognostic factors for cancers, with reduced levels of hemoglobin and RBCs being associated with unfavorable survival outcomes in lung and gastric cancer 26,27. On the contrary, lymphocytes, integral to both the adaptive and innate immune system, play a crucial role in offering antitumor immunity. Specifically, CD4+ and CD8+ T cells identify tumor antigens and have demonstrated their ability to trigger apoptosis in tumor cells 28. Furthermore, tumor biomarkers are uesd as indicators for cancer screening and predictors for therapeutic response and prognoses. Assessing CEA levels has become a valuable complement to tumor detection and staging, recurrence and metastasis monitoring, therapy response and prognosis evaluation in cancer patients^29^,^30^. AFP, a single chain glycoprotein weighing approximately 70 000 Da of molecular weight, has been linked to hepatocellular cancer (HCC) risk^31^, while the biological connection between AFP levels and other cancers requires further investigation. In our study, we found that decreased Hb is an independent prognostic factor in SESCC for predicting DSS, which is consistent with the previous literature^7^. The complex interactions between inflammation biomarkers and esophageal cancer currently are not fully explained and further studies are warranted to examine functional significance of these associations.

We provided SESCC patients with a dynamic prediction model which had a high AUC, stable internal validation and intuitive web version. The indicators included in the prediction model are intuitive and easily available, making the predictive model more applicable in clinical settings. For patients who have predicted poor survival rates after treatment, intensified follow-up may be warranted. There are some previously developed and validated prediction models regarding esophageal cancer (EC)^7^,^32^,^33^. Chen *et al.* constructed a nomogram based on nutritional and inflammatory indicators and the C-index of the nomogram for OS was 0.728, but this nomogram was exclusively designed for survival prediction of small cell carcinoma of the esophagus and only included certain hematological indicators^7^. Liu *et al.* extracted patients diagnosed with EC from the SEER database and builded up nomogram models with the C-index of the OS nomogram being 0.740 (95% CI: 0.707-0.773) and that of the CSS nomogram being 0.752 (95% CI: 0.719-0.785)^32^, which were lower than ours. Novel endoscopic criteria for predicting tumor invasion depth in superficial esophageal squamous carcinoma were proposed with the accuracy being 79.5%^33^, but they used conventional endoscopy alone which needed quite experienced endoscopists to assess and they did not establish a convenient nomogram neither.

In summary, our study has the following advantages: (1) We had an extended follow-up period and relatively complete clinical and pathological data; (2) We enrolled a larger sample size than the previous studies; (3) Innovative hematological biomarkers as predictive factors were also included; (4) We successfully constructed a dynamic web-based prediction model for further validation by more researchers. However, we also had some limitations. Firstly, we were unable to perform external validation. Second, our nomogram showed specificity for superficial esophageal squamous cell carcinoma, and other subtypes of esophageal cancer and other esophageal diseases were not applied in this current study. Furthermore, the procedure duration, hospital stay and hospital cost were not collected. In upcoming studies, we will conduct prospective clinical trials in our center and verify our findings.

## Conclusion

In summary, this long-term cohort study gives a real-world perspective of long-term outcomes of SESCC. Besides, we established a nomogram based on clinicopathological and inflammation-related indicators for predicting DSS and OS in SESCC patients. This model can effectively predict survival risks in patients undergoing treatment for SESCC, thereby enhancing follow-up strategies. Large-scale and multi-center trials are urgently needed to validate our model which might be helpful for clinicians in the treatment and prognostic prediction of SESCC.

## Supplementary Material

Supplementary figures and tables.

## Figures and Tables

**Table 1:** Baseline characteristics of all patients who alive and died of SESCC, cancer characteristics, pathology and outcomes.

	Before matching			After matching		
	Survival	No Survival		Survival	No Survival	
Characteristic	(n=1081)	(n=90)	*P*	(n=90)	(n=90)	*P*
Age over 65, n (%)	491 (45.4)	34 (37.8)	0.197	31 (34.4)	34 (37.8)	0.642
Male, n (%)	764 (70.7)	70 (77.8)	0.191	64 (71.1)	70 (77.8)	0.305
ESD, n (%)	719 (66.5)	18 (20.0)	<0.001	18 (20.0)	18 (20.0)	1.000
Smoke, n (%)	434 (40.2)	43 (47.8)	0.192	50 (55.6)	43 (47.8)	0.296
Alcohol, n (%)	350 (32.4)	27 (30.0)	0.730	28 (31.1)	27 (30.0)	0.871
CCI, n (%)			0.002			0.135
0	717 (66.3)	52 (57.8)		65 (72.2)	52 (57.8)	
1	302 (28.0)	31 (34.4)		19 (21.1)	31 (34.4)	
2	58 (5.4)	4 (4.4)		5 (5.6)	4 (4.4)	
3	4 (0.4)	3 (3.3)		1 (1.1)	3 (3.3)	
Lesion location, n (%)			0.004			0.624
Upper	130 (12.0)	8 (8.9)		11 (12.2)	8 (8.9)	
Middle	351 (32.5)	27 (30.0)		30 (33.3)	27 (30.0)	
Lower	596 (55.1)	52 (57.8)		48 (53.3)	52 (57.8)	
Whole	4 (0.4)	3 (3.3)		1 (1.1)	3 (3.3)	
Depth of invasion, n (%)			<0.001			<0.001
M1	545 (50.4)	7 (7.8)		22 (24.4)	7 (7.8)	
M2	104 (10.0)	1 (1.1)		6 (6.7)	1 (1.1)	
M3	171 (15.8)	15 (16.7)		20 (22.2)	15 (16.7)	
SM	261 (24.1)	67 (74.4)		42 (46.7)	67 (74.4)	
Lesion diameter (cm), M (P25, P75)	2.6 (1.9, 3.1)	2.3 (1.5, 3.0)	1	2.1 (1.2, 3.0)	2.3 (1.5, 3.0)	0.180
Differentiation, n (%)			<0.001			<0.001
Well	429 (39.7)	2 (2.2)		18 (20.2)	2 (2.2)	
Middle	505 (46.7)	37 (41.1)		50 (55.6)	37 (41.1)	
Poor	147 (13.6)	51 (56.7)		22 (24.4)	51 (56.7)	
Lymphovascular invasion, n (%)	58 (5.4)	30 (33.3)	<0.001	3 (3.3)	30 (33.3)	<0.001
LNM, n (%)	129 (11.9)	42 (46.7)	<0.001	11 (12.2)	42 (46.7)	<0.001
Additional treatment, n (%)	113 (10.5)	11 (12.2)	0.730	6 (6.7)	11 (12.2)	0.203
Recurrence/metastasis, n (%)	50 (4.6)	9 (10.0)	0.047	2 (2.2)	9 (10.0)	0.029
Survival time (month), M (P25, P75)	68.6 (41.0, 93.0)	42.8 (23.5, 58.3)	<0.001	83.9 (66.5, 113.5)	42.8 (23.5, 58.3)	<0.001

Notes: P-values were determined using the Mann-Whitney U-test and χ^2^ test. ESD = endoscopic submucosal dissection; SESCC = superficial esophageal squamous cell carcinoma; CCI = Charlson Comorbidity Index; LNM = lymph node metastasis.
