# Supplementary material for: A Long-term Survival Risk Prediction Model for Patients with Superficial Esophageal Squamous Cell Carcinoma
Source: J Cancer. 2024 Oct 14;15(19):6204–12. doi: 10.7150/jca.99042 (PMC11540495; doi:10.7150/jca.99042)
Supplement: Supplementary file 1 — Supplementary figures and tables. [file jcav15p6204s1.pdf]

Supplementary document

For predicting OS, it was found whole location, SM, poorly differentiated tumor, LVI, LNM, lymphocyte higher than 2.2x10^{9}/L, neutrophils higher than 66.6x10^{9}/L, RBC lower than or equal to 3.27x10^{12}/L, and CEA over 2.09 U/ml significantly increased the risk of death autonomously.

Supplementary Table 1: Lasso regression and multivariate Cox regression analyses for screening predictors for DSS.

Variables	Lasso regression of DSS lambda.1se	Multivariate Cox analysis of DSS HR (95%CI)	*P*
Gender			
Male	-0.20836029	Ref	
Female		0.67 (0.4-1.12)	0.127
CCI			
0		Ref	
1	0.09712693	1.89(1.2-3)	0.006
2		0.88(0.31-2.45)	0.803
3		1.89(0.51-7.09)	0.344
Differentiation			
Well	0.76257109	Ref	
Middle		7.21(1.66-31.24)	0.008
Poor		16.34(3.76-71.08)	<0.001
LVI	1.05546262		
No		Ref	<0.001
Yes		3.63(2.08-6.33)	
LNM			
No	0.93024164	Ref	
Yes		3.10(1.92-5)	<0.001
Additional treatment			
No	0.30261027	Ref	
Yes		2.58(1.29-5.14)	0.007
Neutrophils			
≤32.2	0.42365338	Ref	
>32.2		2.59(1.39-4.82)	0.003
RBC			
<4.45	-1.00336722	Ref	
≥4.45		0.15(0.07-0.32)	<0.001
Hb			
≤98	-0.92078182	Ref	
>98		0.25(0.08-0.83)	0.023
AFP			
≤3.24	0.37662764	Ref	
>3.24		1.74 (1.04-2.9)	0.034

Supplementary Table 2: Univariate and multivariate Cox regression analyses for screening predictors for OS.

Variables	Univariate Cox analysis of OS HR (95%CI)	*P*	Multivariate Cox analysis of OS HR (95%CI)	*P*
Primary site				
Whole	Ref		Ref	
Lower	0.20(0.06-0.07)	0.005	0.46(0.15-1.47)	0.191
Middle	0.17(0.08-0.61)	<0.001	0.20 (0.07-0.6)	0.004
Upper	0.22(0.48-0.61)	0.003	0.33 (0.11-0.92)	0.035
Invasion depth				
M1	Ref		Ref	
M2	0.22(0.03-1.92)	0.145	0.27(0.04-2.01)	0.200
M3	3.36(1.67-3.32)	<0.001	1.69(0.95-3.03)	0.077
SM	5.33(5.88-8.55)	<0.001	1.43(0.82-2.48)	0.210
Differentiation				
Well	Ref		Ref	
Middle	8.33(3.31-16.54)	<0.001	5.05(1.9-13.45)	0.001
Poor	40.86(20.95-100.9)	<0.001	10.45(3.93-27.74)	<0.001
Lymphovascular invasion				
No	Ref		Ref	
Yes	12.72(8.97-18.03)	<0.001	3.88(2.31-6.49)	<0.001
LNM				
No	Ref		Ref	
Yes	8.41(5.98-11.84)	<0.001	3.78(2.4-5.96)	<0.001
Lymphocyte				
≤2.28	Ref		Ref	
>2.28	2.18(1.52-3.11)	<0.001	2.90(1.62-5.17)	<0.001
Neutrophils				
≤66.6	Ref		Ref	
>66.6	2.18(1.48-3.21)	<0.001	2.17(1.45-3.23)	<0.001
RBC				
≤3.27	Ref		Ref	
>3.27	0.1(0.04-0.26)	<0.001	0.09(0.03-0.28)	<0.001
CEA				
≤2.09	Ref		Ref	
>2.09	2.47(1.53-3.98)	<0.001	1.63(0.99-2.69)	0.054

Supplementary Figure 1: Nomogram for the early prediction the overall survival probability. Characteristics in the nomogram to predict probability of overall survival. To use the nomogram,

the specifific points of individual patients are located on each variable axis. Lines and dots are drawn upward to determine the points received by each variable; the sum of these points is located on the Total Points axis, and a line is drawn downward to the "1-year survival, 3-year survival and 5-year survival" axes to determine the probability of overall survival. LNM = lymph node metastasis; RBC = red blood cell; CEA = carcinoembryonic antigen.

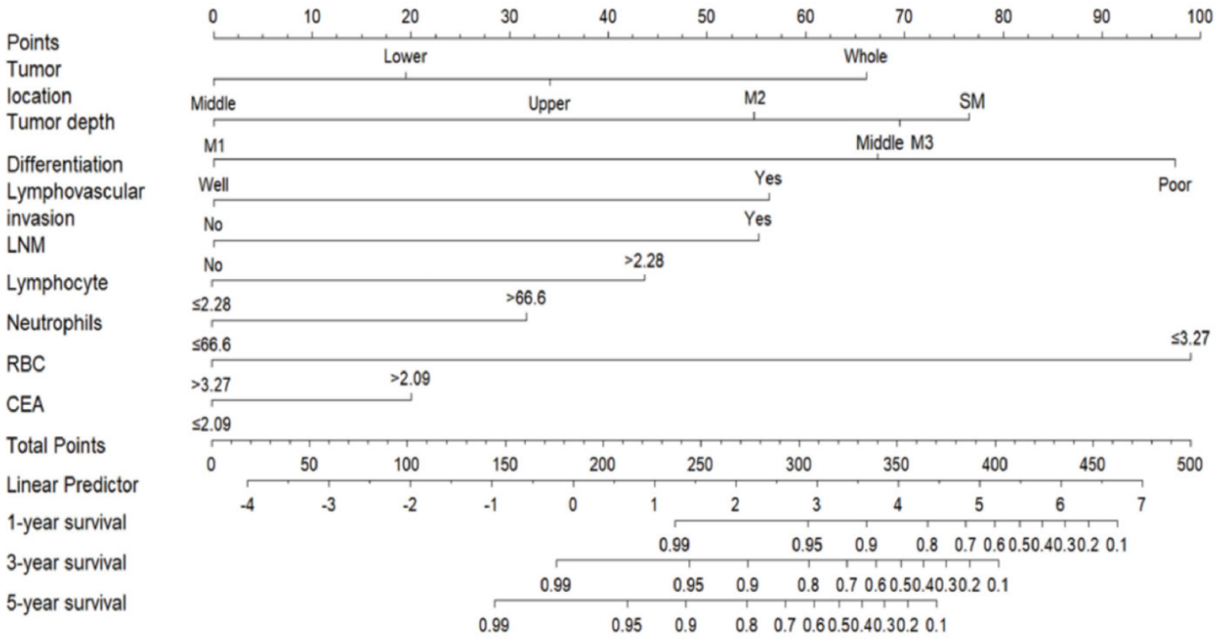

Supplmental Figure 2: ROC curve, AUC (A), and calibration curve (B) for OS of the nomogram. ROC = receiver operating characteristic; AUC = the area under ROC curve; OS = overall survival. Calibration curves of 5 years overall survival.

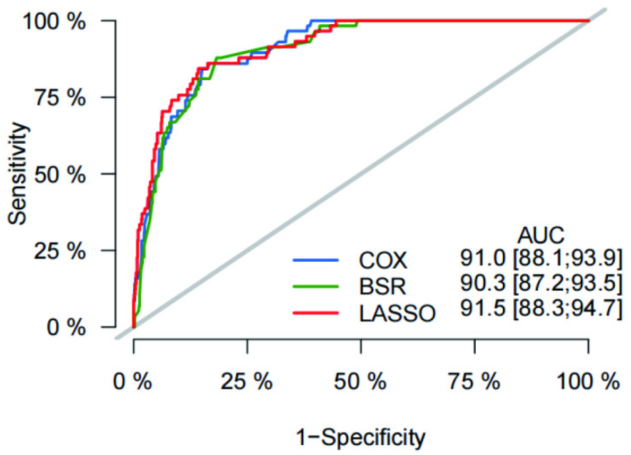

A

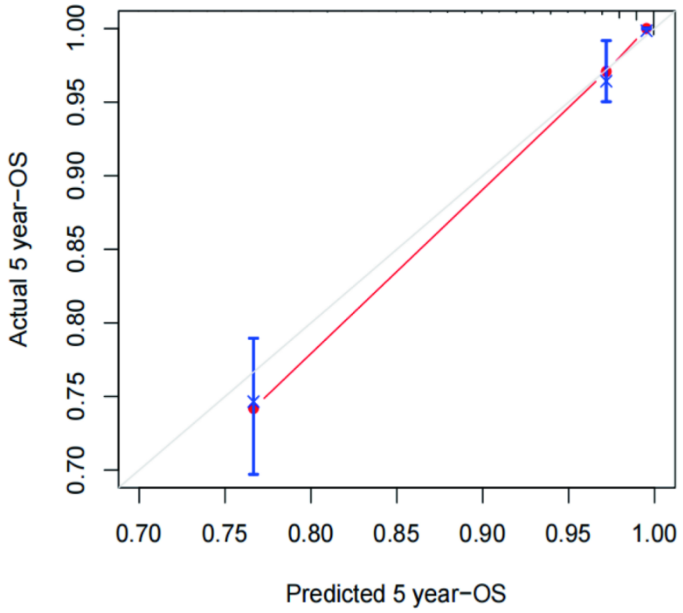

B

Supplmental Figure 3: Internal validation of SESCC nomogram available on the Internet by DSS (A) and OS (B).

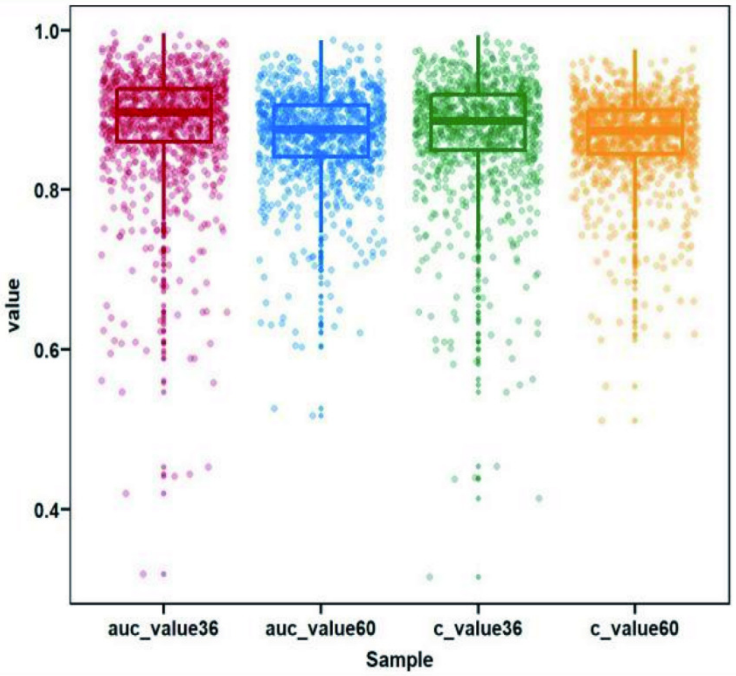

A

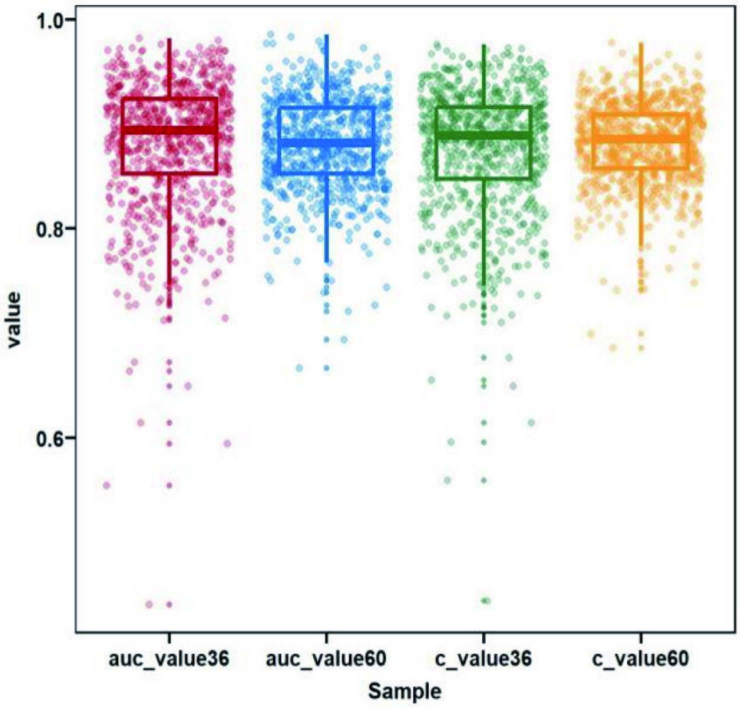

1.0
0.8
0.6
value
auc_value36
auc_value60
c_value36
c_value60
Sample

B
